# Supplementary material for: COVID-19 related concerns of people with long-term respiratory conditions: a qualitative study
Source: BMC Pulm Med. 2020 Dec 9;20:319. doi: 10.1186/s12890-020-01363-9 (PMC7724437; doi:10.1186/s12890-020-01363-9)
Supplement: Supplementary file 1 — Additional file 1. Full survey questions and possible responses. [file 12890_2020_1363_MOESM1_ESM.docx]

**COVID-19 RELATED CONCERNS OF PEOPLE WITH LONG-TERM RESPIRATORY CONDITIONS: A QUALITATIVE STUDY – SUPPLEMENT 1**

**FULL SURVEY QUESTIONS AND POSSIBLE RESPONSES:**

**Free text question used for current study:**

- What are your main concerns about getting coronavirus?

**Additional survey questions** **(not analysed in the current study)**

- What is your age? 17 and under; 18-29; 30-39; 40-49; 50-59; 60-69; 70-79; 80+
- What is your gender? Male; Female; Other
- What nation do you live in? England; Northern Ireland; Scotland; Wales
- Have you ever been told that you have 'difficult', 'brittle' or 'severe' asthma? (AUK survey only) Yes; No; Not sure What is your main lung diagnosis? Asthma; COPD (chronic obstructive pulmonary disease); Bronchiectasis; ILD (interstitial lung disease); I do not have a lung condition; Other
- Do you receive pulmonary rehabilitation as part of your care? Yes; No.
- When do you get out of breath? (MRC dyspnoea scale options) (BLF survey only) i) I'm not troubled by being out of breath, except on strenuous exercise; ii) I'm short of breath when hurrying on the level or walking up a slight hill; iii) I walk slower than most people on the level, stop after a mile or so, or stop after 15 minutes of walking at my own pace; iv) I stop for breath after walking about 100 yards or after a few minutes on level ground; v) I'm too breathless to leave the house, or breathless when dressing and undressing
- Are you currently following the government's 'shielding' advice? Yes; No.
- Have you received a letter or text message advising you to shield? Yes; No; Don’t know.
- Did you have the flu jab this winter? Yes; No; Don’t know.
- Have you seen guidance or advice on coronavirus from Asthma UK or the British Lung Foundation? Yes; No.
- How did you see this advice? Via email; On Twitter; On Facebook; On the AUK and/or BLF websites.
- Where do you mainly get your information about coronavirus? TV; Radio; News websites; AUK / BLF websites; AUK /BLF social media; Other social media; Friends and family.
- What was it about the advice you saw that wasn't helpful? It was too complicated; There was not enough detail; I didn't trust it; It was badly presented; It couldn't answer my query.
- The Government? Very helpful; Helpful; Neither helpful nor unhelpful; Not helpful; Not helpful at all; I have not seen their advice.
- What was it about the advice you saw that wasn't helpful? It was too complicated; There was not enough detail; I didn't trust it; It was badly presented; It couldn't answer my query
- Would you like more information on any of the following during the coronavirus outbreak? Yes, No.
- Are you currently suffering from any coronavirus symptoms? Yes; No; Not sure.
- Are you following isolation advice? Yes; No.
- How confident are you about following advice to self-isolate or shield? Very confident; Somewhat confident; Neither confident nor not confident; Not very confident; Not confident at all.
- How confident are you that you will be able to access food and other provisions if you have to self isolate or shield? Very confident; Somewhat confident; Neither confident nor not confident; Not very confident; Not confident at all.
- Do you have enough medicines for your needs at the moment? Yes; No.
- Are you registered to order prescriptions online from your GP? Yes; No.
- Have you had a look at online inhaler technique videos to check that you are using your inhalers properly? Yes; No; I don’t use inhalers.
- Do you have a written self-management plan for your condition? Yes; No.
- In general, how well prepared do you feel for coronavirus? 1-10, with 10 being most prepared
- In general, how anxious do you feel about coronavirus? 1-10, with 10 being most anxious
- Which of the following apply to you? I'm concerned about my lung condition; I'm concerned about the health of my family members; I'm coping well; I'm having trouble getting groceries; I'm worried about my financial situation due to missed work; I can't get the prescriptions I need; None of the above; Other
- Have you used any of these services due to coronavirus, or concerns about its symptoms? NHS 111 (online checker); NHS 111 (telephone advice); GP; Pharmacy; A&E; BLF or AUK nurse phone; Other; Has any of the following happened to you because of the way the NHS is working at the moment?; I have had to have a GP appointment conducted over the phone / remotely; My regular care for my lung condition at the hospital has been cancelled; My regular care for my lung condition at the GP has been cancelled; Pulmonary rehabilitation classes were cancelled; I have had to do pulmonary rehabilitation exercises at home; None of the above; Other
- Are you able to perform your job at home? Yes; No; I’m not in work.
- How supportive have your workplace been during the coronavirus outbreak? Very supportive; Supportive; Somewhat supportive; Not supportive; Not supportive at all.
- Are you keeping physically active, or are able to do any exercise at home? Yes; No.
- Which of the following are you doing to stay active while at home? Housework; Gardening; Going on walks; Yoga; Cycling; Running; Other
- If you smoke, are you planning to try to quit smoking to protect yourself from coronavirus? Yes; No; I don't smoke.
- Are you actively self-isolating due to coronavirus? Yes; No.
- Do you live alone? Yes; No.
- How often do you feel you lack companionship? Hardly ever; Some of the time; Often.
- How often do you feel left out of things? Hardly ever; Some of the time; Often.
- How often do you feel isolated from others? Hardly ever; Some of the time; Often.
- What do you think about measures (such as social distancing and lockdown) to protect yourself during coronavirus?
- What good advice do you have (or have you heard) about coping with coronavirus that you would like to share?
